# Red recombination enables a wide variety of markerless manipulation of porcine epidemic diarrhea virus genome to generate recombinant virus

**DOI:** 10.3389/fcimb.2023.1338740

**Published:** 2024-01-22

**Authors:** Shuonan Pan, Chunxiao Mou, Zhenhai Chen

**Affiliations:** ^1^ College of Veterinary Medicine, Yangzhou University, Yangzhou, China; ^2^ Jiangsu Co-Innovation Center for Prevention and Control of Important Animal Infectious Diseases and Zoonoses, Yangzhou University, Yangzhou, China; ^3^ Jiangsu Key Laboratory of Zoonosis, Yangzhou University, Yangzhou, China; ^4^ Joint International Research Laboratory of Agriculture and Agri-Product Safety, the Ministry of Education of China, Yangzhou University, Yangzhou, China

**Keywords:** porcine epidemic diarrhea virus, reporter virus, ORF3, RED recombination, antiviral

## Abstract

Porcine epidemic diarrhea virus (PEDV) is a member of the genera *Alphacoronavirus* that has been associated with acute watery diarrhea and vomiting in swine. Unfortunately, no effective vaccines and antiviral drugs for PEDV are currently available. Reverse genetics systems are crucial tools for these researches. Here, a PEDV full-length cDNA clone was constructed. Furtherly, three PEDV reporter virus plasmids containing red fluorescent protein (RFP), Nano luciferase (Nluc), or green fluorescence protein (GFP) were generated using Red recombination with the GS1783 *E. coli* strain. These reporter-expressing recombinant (r) PEDVs showed similar growth properties to the rPEDV, and the foreign genes were stable to culture up to P9 in Vero cells. Using the Nluc-expressing rPEDV, the replication of PEDV was easily quantified, and a platform for rapid anti-PEDV drug screening was constructed. Among the three drugs, Bergenin, Umifenovir hydrochloride (Arbidol), and *Ganoderma lucidum* triterpenoids (GLTs), we found that GLTs inhibited PEDV replication mainly after the stage of virus “Entry”. Overall, this study will broaden insight into the method for manipulating the PEDV genome and provide a powerful tool for screening anti-PEDV agents.

## 1 Introduction

Coronaviruses (CoVs; order *Nidovirales*, family *Coronaviridae*, subfamily *Orthocoronavirinae*) cause respiratory and intestinal infections in animals and humans (Cui et al., 2019). The family Coronaviridae comprises four genera: *Alphacoronavirus*, *Betacoronavirus*, *Gammacoronavirus*, and *Deltacoronavirus* (Jung et al., 2020). Porcine epidemic diarrhea (PED) is a highly contagious swine disease caused by porcine epidemic diarrhea virus (PEDV), an *Alphacoronavirus* that leads to acute watery diarrhea and vomiting. PEDV was first reported in the early 1970s in the United Kingdom and has gradually spread throughout Europe, North America, and Asia (Wood, 1977; Takahashi et al., 1983; Pijpers et al., 1993; Nagy et al., 1996; Pritchard et al., 1999; Li et al., 2012). PEDV strains were usually classified into two genotypes, G1 and G2. PEDV strains displayed a high degree of gene variation since their emergence in China in 2010 (Lee et al., 2010; Li et al., 2012). Specifically, the G2 strain can be further divided into four subgroups: G2a, G2b, G2c, and G2d (Huang et al., 2013; Tan et al., 2020). From 2017 to 2021, the PEDV G2c subgroup is the predominant lineage circulating in China swine farms, causing significant economic challenges for the swine industry (Huang et al., 2013).

PEDV is a single-stranded, positive-sense RNA virus. The genome measures approximately 28 kb nucleotides (nts) and consists of several components including the following: a 5’ untranslated region (UTR), open reading frame (ORF) 1a and ORF 1b, spike (S), ORF3, envelope (E), membrane (M), and nucleocapsid (N) genes, and a 3’UTR. The two large ORFs encode two polyproteins, pp1a and pp1b, which are cleaved post-translationally to produce 16 non-structural proteins (nsps) known as nsp1-nsp16. Four structural proteins are encoded by the S, E, M, and N genes. Additionally, an accessory protein is expressed by the ORF3 gene located between the S and E genes (Zhang et al., 2022b). ORF3 has been reported to have varying degrees of deletion in the PEDV field strains and to be not essential for virus replication, allowing it to be replaced by foreign genes using reverse genetics systems (Park et al., 2008).

Reverse genetics systems are crucial tools to study the molecular biology and pathogenesis of viruses, to research viral gene functions, to investigate virus-host interactions, and to develop antiviral drugs (Fan et al., 2017; Peng et al., 2020). The coronavirus genome cloned in a high-copy vector in *E. coli* is often unstable due to its toxicity, the BAC system was therefore preferably used to construct coronavirus infectious clone (Fan et al., 2017; Peng et al., 2020; Zhang et al., 2020). To create PEDV mutants using infectious cDNA clone *in vitro*, clustered regularly interspaced palindromic repeats (CRISPR)/CRISPR-associated protein 9 (Cas9) (CRISPR/Cas9) technology (Peng et al., 2020; Zhou et al., 2022) and the method of restriction enzyme digestion and ligation were usually carried out (Beall et al., 2016). Both of the above methods require either a high concentration of the infectious plasmids or subclone plasmids, which creates a great inconvenience for the experiments. To circumvent the challenges associated with manipulating the PEDV infectious cDNA clone, we employed the Red recombination method using *E. coli* strain GS1783, which is equipped with an inducible Red and I-SceI-expression encoded on the chromosome (Tischer et al., 2010).

In this study, based on the full-length PEDV strain GX4/2021 cDNA clone, three PEDV reporter viruses expressing the Nano luciferase (Nluc), red fluorescent protein (RFP), or green fluorescent protein (GFP) were generated by Red recombination with the *E. coli* strain GS1783. The expression of the three reporter genes was stable up to nine passages in virus-infected Vero cells. Moreover, recombinant (r) PEDV-Nluc/ORF3 can be applied to screening antiviral drugs. *Ganoderma lucidum* triterpenoids (GLTs) was found to inhibit PEDV replication mainly after the stage of virus “Entry”. This study will broaden insight into the method for manipulating the PEDV genome, and develop a reliable method for screening anti-PEDV reagents.

## 2 Materials and methods

### 2.1 Cells, viruses, and antibodies

African green monkey kidney epithelial (Vero) cells were cultured in Dulbecco’s modified Eagle’s medium (DMEM) (Gibco, USA) containing 10% fetal bovine serum (FBS) (Sigma, USA) and 1% penicillin-streptomycin (Solarbio, China). The wild-type PEDV strain GX4/2021 (GenBank accession number OP382083) was grown on Vero cells using a maintenance medium, DMEM supplemented with 2 μg/ml of trypsin (Gibco, USA), 0.3% tryptose phosphate broth (TPB) (Sigma, USA), and 1% penicillin-streptomycin. A monoclonal antibody (mAb) against PEDV N protein was produced in mice, and a polyclonal antibody (pAb) against glyceraldehyde-3-phosphate dehydrogenase (GAPDH) was purchased from Proteintech, China, and a mAb against beta actin was purchased from Huabio, China.

### 2.2 Construction of a PEDV full-length infectious clone

**Figure 1:**
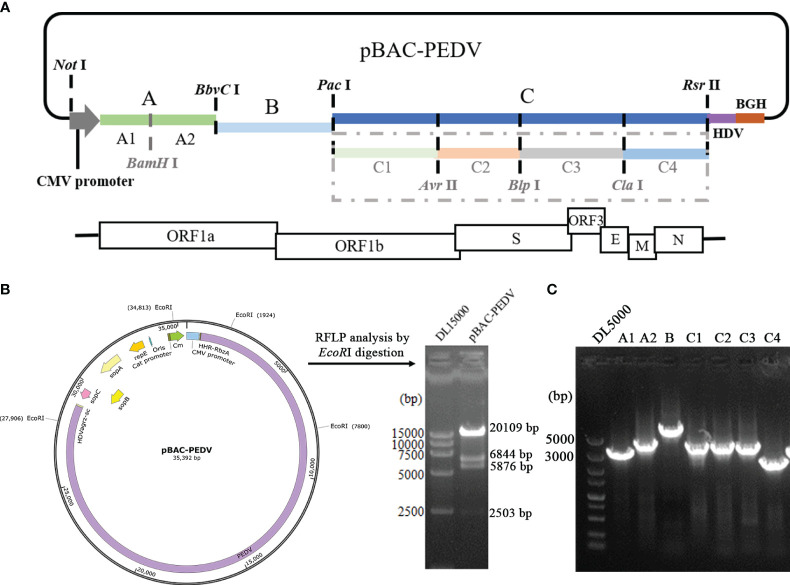
Construction of a PEDV infectious clone. **(A)** Schematic diagram depicting the construction of a PEDV full-length cDNA clone of the GX4/2021 strain. Restriction enzyme sites in the PEDV genome were employed to ligate the full-length PEDV GX4/2021 genome. **(B)** Restriction fragment length polymorphism analysis with *EcoR*I restriction digestion of pBAC-PEDV. Sizes of the digested bands are displayed. **(C)** Identification of pBAC-PEDV by PCR with primers in **Table 1**.

The PEDV full-length cDNA is assembled into pBeloBAC11 vector. The restriction sites, namely *Bam*HI, *Bbvc*I, *Pac*I, *Avr*II, *Blp*I, *Cla*I, and *Sac*II, which naturally occurred in the PEDV-GX4/2021 genome, were selected. The synthesis of PEDV cDNA was carried out by Hifair^®^ AdvanceFast 1st Strand cDNA Synthesis Kit (Yeasen Biotechnology, China). Seven overlapping DNA fragments (A1, A2, B, C1, C2, C3, C4) were amplified by PCR using PrimeSTAR^®^ Max DNA Polymerase (Takara, China) and the primers were listed in **Table 1**. A CMV promoter, a 35-residue poly (A) tail, hepatitis delta virus (HDV) ribozyme sequence and bovine growth hormone (BGH) termination signal were inserted into the PEDV infectious clone. Finally, all the fragments were ligated into the linearized vector by restriction enzyme sites as shown in **Figure 1A**. The PEDV infectious clone plasmid is named pBAC-PEDV.

**Table 1:** Primers used for the construction of PEDV full-length cDNA clone.

Primer	Primer Sequence (5’-3’)
PEDV-A1 F	CGAAACGGAGTCTAGACTCCGTCACTTAAAGAGATTTTCTATCTATGG
PEDV-A1 R	GTCAAAAGCAAAAGGATCCTTAGTAACTGTGGA
PEDV-A2 F	CAGTAGTGCGGCCGCCAGTTACTAAGGATCCTTTTGCTTTTGACTTTGC
PEDV-A2 R	AACGTACGCCTCAGCAACAGCAGCATTAAAGG
PEDV-B F	CTGCTGTTGCTGAGGCTCATCGTTACG
PEDV-B R	CAAGCGCCTACCTTAATTAAAATGCTC
PEDV-C1 F	TAAGAGCATTTTAATTAAGGTAGGCGCTTG
PEDV-C1 R	CAGCCTTTGACCACCTAGGATTTTTAGCC
PEDV-C2 F	GGCTAAAAATCCTAGGTGGTCAAAGGCTG
PEDV-C2 R	AGTGTTAGCTGAGCACCTGGTGACATC
PEDV-C3 F	GATGTCACCAGGTGCTCAGCTAACACT
PEDV-C3 R	GTGTTTTGTTAACATCGATGTAATCCGGG
PEDV-C4 F	CCCGGATTACATCGATGTTAACAAAACAC
PEDV-C4 R	AGGTCGGACCGCGAGGAGGTGGAG

### 2.3 Generation of recombinant virus expressing exogenous genes with Red recombination

**Figure 2:**
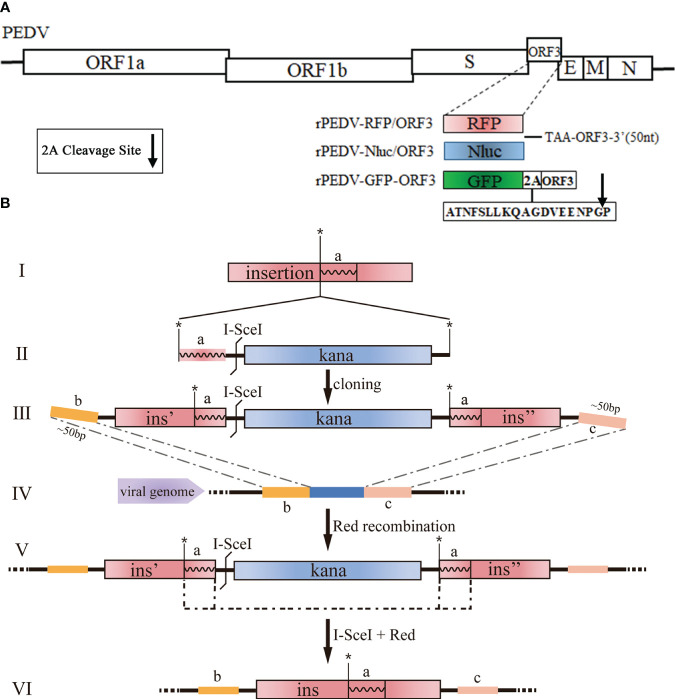
Generation of recombinant viruses expressing foreign genes with the *E. coli* strain GS1783. **(A)** Schematic diagram depicting the insertion of RFP, Nluc, and GFP into PEDV genome. Arrow represents the 2A cleavage site. **(B)** Schematic diagram depicting the Red recombination using the Red recombination. *: an indicator for second recombine; a, b, c: homologous arms; ins, ins', ins": sequence or portions of the sequence that shall be inserted.

The cDNA clone plasmids containing Nluc, RFP, or GFP (**Figure 2A**) were produced with Red recombination using the *E. coli* strain GS1783. As shown in **Figure 2B**, the en passant marker cassette containing a short duplication sequence of insertion (ins, the fragment a), *I-Sce*I, and kanamycin (Kana) is amplified by PCR using primers (**Figure 2B** II). The universal transfer construct ins-Kana containing the 40-50 bases homologous arms for the PEDV genome was amplified by overlap PCR (**Figure 2B** III). The universal transfer ins-Kana was electroporated into the GS1783 *E. coli* containing the full-length PEDV infectious clone plasmid for the first Red recombination (**Figure 2B** IV). The positive colonies in the Red recombination could grow on an LB agar plate containing Kana (30 μg/ml) and chloramphenicol (CAP, 34 μg/ml). Finally, the marker cassette is deleted from the intermediate (**Figure 2** V) by the *I-Sce*I site cleavage through the induction of L-arabinose *in vivo* for a second red recombination (**Figure 2** VI). The primers used above were described in **Table S1** in the **Supplementary Material**.

### 2.4 Recovery of recombinant viruses

In a 6-well culture plate, Vero cells with 70% confluence were co-transfected with 1.8μg full-length cDNA clone plasmid (pBAC-PEDV, pBAC-PEDV-RFP/ORF3, pBAC-PEDV-Nluc/ORF3, or pBAC-PEDV-GFP-ORF3), and 0.2μg pCAGGS-PEDV-N using Lipofectamine™ 3000 reagent (Invitrogen, USA). After 24 hours of transfection, the cell monolayers were washed with phosphate-buffered saline (PBS) and treated with a maintenance medium. The cells were then inoculated at 37°C. The cytopathic effect (CPE) and red or green fluorescence were observed daily with a fluorescence microscope (Leica, Germany).

### 2.5 Indirect immunofluorescence assay

In a 12-well culture plate, Vero cells were washed twice with PBS and infected with the indicated viruses at a multiplicity of infection (MOI) of 0.001 for 24h. The cell monolayers were fixed with 4% paraformaldehyde for 10 min. After washed with PBS, the cells were permeabilized using 0.1% Triton X-100 and blocked with 2% bovine serum albumin (BSA) in PBS for 30 min at room temperature. The cell monolayers were incubated with mAb PEDV N for 1 h at 37°C and further stained with Dylight 488-labeled goat anti-mouse IgG (H+L) (Abbkine, China). Then the cell nuclei were stained with 2 μg/ml 4′,6-diamidino-2-phenylindole (DAPI) (Solarbio, China) for 10 min. After washing twice with PBS, fluorescent pictures were taken using a fluorescence microscope (Leica, Germany).

### 2.6 Western blot analysis

The Vero cells were lysed in RIPA Lysis Buffer (Beyotime, China). The cell lysates were mixed with 5× sodium dodecyl sulfate (SDS) loading buffer and heated at 98°C for 5 min. The proteins were separated through 12% SDS-polyacrylamide gels and were subsequently transferred onto polyvinylidene difluoride (PVDF) membranes. The membranes were blocked with 5% skimmed milk in TBST (20 mM Tris, 150 mM NaCl, 0.1% Tween 20) at room temperature for 2 hours. Then, the membranes were incubated overnight at 4°C with mAb against PEDV N and pAb against GAPDH. The membranes were washed three times with TBST and then were incubated with HRP-conjugated secondary antibodies. Finally, the membranes were visualized using NcmECL Ultra (NCM Biotech, China) by a chemiluminescence imaging system (Tanon, China).

### 2.7 Viral growth curves

In a 24-well (10^5^ cells/well) plate, Vero cells were infected with recombinant viruses at 0.001 MOIs. After incubation for 2 h, the cell supernatants were removed and a maintenance medium was added to the cell monolayers. The cell culture supernatants were collected at different time points. The virus titers were determined by a TCID_50_ assay following the Karber method. The viral growth curves were made by using GraphPad Prism software (version 8.0).

### 2.8 Plaque assay

In a 12-well culture plate, recombinant viruses were introduced to confluent Vero cells at a multiplicity of infection (MOI) of 0.0001. Following a 2-hour incubation, the cell culture supernatants were aspirated. Subsequently, the cells were washed twice with PBS, and a maintenance medium supplemented with 1% low melting point agarose was applied to the cell monolayers. At 36 hours post-infection (hpi), the cells were fixed and stained using a solution of 20% alcohol and 1% crystal violet in physiological saline.

### 2.9 Luciferase activity assay

In a 96-well culture plate, Vero cells were infected with the indicated viruses in triplicate wells. Samples were collected at 3, 6, 9, and 12 hpi, respectively. Following the manufacturer’s instructions, the luciferase activity was then measured with Nano-Glo® Luciferase Assay System (Promega, USA).

### 2.10 Genetic stability of foreign gene in the PEDV reporter virus

PEDV reporter viruses were passaged nine times (P1 to P9) in Vero cells and the viral RNAs of P3 and P9 were extracted. Then the exogenous genes were identified by RT-PCR and DNA sequencing.

### 2.11 Cell viability assay

After Vero cells and ST cells were treated with the indicated drugs, the cell viability was assessed using the Cell Counting Kit-8 (CCK-8, Yeasen Biotechnology, China). In a 96-well cell plate, the single layers were washed twice with PBS, followed by incubation at different concentrations of drugs or DMSO. CCK-8 solution (10 μL/well) was added to the plate at the indicated time. After incubation for 3 h at 37°C, the absorbance was tested at the 450 nm wavelength.

### 2.12 Total RNA extraction and quantification

Total RNA was extracted from the cells using RNA-easy Isolation Reagent (Vazyme Biotech, China), followed by reverse transcription of the RNAs into cDNA using Hifair^®^ V one-step RT-gDNA digestion SuperMix (Yeasen Biotechnology, China). The primers targeting PEDV N gene, monkey GAPDH, and pig GAPDH were designed in **Table 2**. Gene expression was estimated using Hieff^®^ qPCR SYBR Green Master Mix (Yeasen Biotechnology, China) by a LineGene 9600 system (Bioer Technology, China). Relative expression levels of PEDV N were calculated with the 2^-ΔΔCt^ method.

**Table 2:** Primers used for real-time RT-PCR assay.

q-PEDV-N F	CCGTGGTGAGCGAATTGAAC
q-PEDV-N R	TCAGACGCCTTTCTGACACC
q-pig-GAPDH F	TCATCATCTCTGCCCCTTCT
q-pig-GAPDH R	GTCATGAGTCCCTCCACGAT
q-monkey-GAPDH F	TCGGAGTCAACGGATTTGGT
q-monkey-GAPDH R	AACCTGGGGGAATACGCAAG

### 2.13 Statistical analysis

The experimental groups were compared to the mock-treated group using one-way ANOVA in GraphPad Prism (version 8.0) software. The levels of statistical significance were defined as follows: *, p < 0.05; **, p < 0.01; ***, p < 0.001; ****, p < 0.0001.

## 3 Results

### 3.1 Construction of the PEDV full-length cDNA clone

To construct the PEDV full-length cDNA clone, a set of fragments covering the entire PEDV-GX4/2021 genome were assembled into pBeloBAC11 (**Figure 1A**). The PEDV infectious clone plasmid was named pBAC-PEDV. The CMV promoter was inserted at the 5’ terminus of virus genome, and a 35-residue poly (A) tail was placed at 3’end. The complete cDNA clone plasmid was digested with *EcoR*I to perform restriction fragment length polymorphism (RLFP) analysis, which produced four specific fragments as expected (**Figure 1B**). Meanwhile, the pBAC-PEDV was detected using PCR with the primers described in **Table 1** (**Figure 1C**) and verified by full-length nucleotide sequencing. Taken together, we successfully constructed the PEDV full-length cDNA clone plasmid.

### 3.2 Recovery and Identification of rPEDV

**Figure 3:**
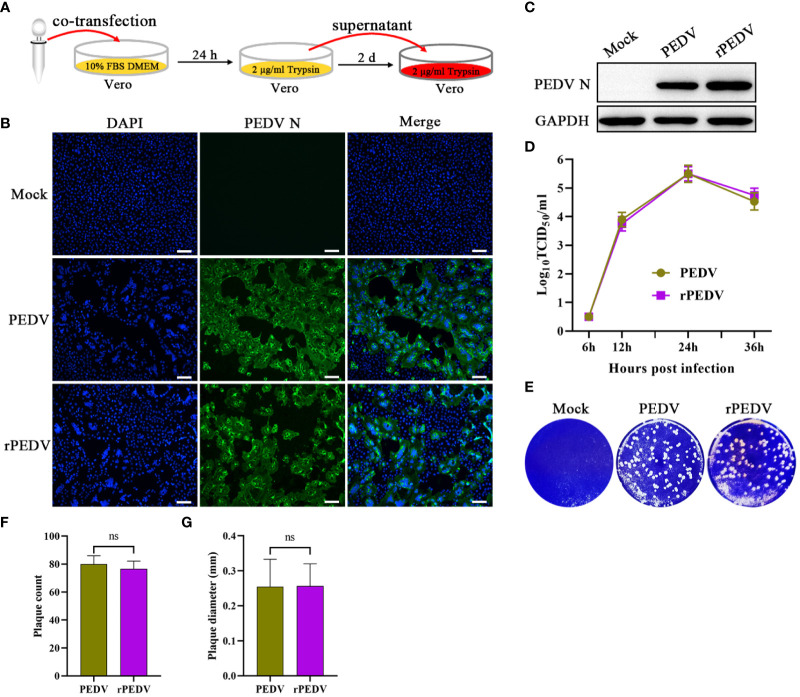
Recovery and identification of rPEDV. **(A)** Schematic diagram of rescuing and passaging rPEDV on Vero cells. **(B)** IFA and **(C)** western blot of mock-, parental PEDV strain-, or rPEDV-infected Vero cells at 24 hpi using anti-PEDV N monoclonal antibody. The bar=100 μm. **(D)** Multiple-step growth curves of PEDV and rPEDV on Vero cells. Cells were infected with PEDV or rPEDV at an MOI of 0.001. The cell supernatants were collected at 6, 12, 24, and 36 hpi, respectively. The virus titers were determined using a TCID_50_ assay. **(E)** Plaque assay. The confluent Vero cells were infected with PEDV or rPEDV at 0.0001MOIs. At 30 hpi, the cells were stained with 1% crystal violet. **(F)** Average plaque count and **(G)** average plaque diameter for PEDV and rPEDV. P-values were determined by one-way ANOVA. ns, no significant.

According to the strategy outlined in **Figure 3A**, the supernatant of the transfected cells was further passaged onto Vero cells to generate rPEDV. In accordance with wild-type PEDV, the expression of PEDV N in rPEDV-infected cells could also be detected by IFA (**Figure 3B**) and western blot (**Figure 3C**). To investigate the growth characteristics of rPEDV in relation to wild type PEDV, the viral growth curves and plaque size were detected. As shown in **Figure 3D**, the rescued rPEDV showed similar growth kinetic to the wild-type PEDV, with their titers peaking at 24 hpi (10^5.5^TCID_50_/mL). There is also no significant difference between the rPEDV and wild-type PEDV as confirmed by plaque assay (**Figures 3E–G**).

### 3.3 Construction of rPEDV expressing reporter genes

**Figure 4:**
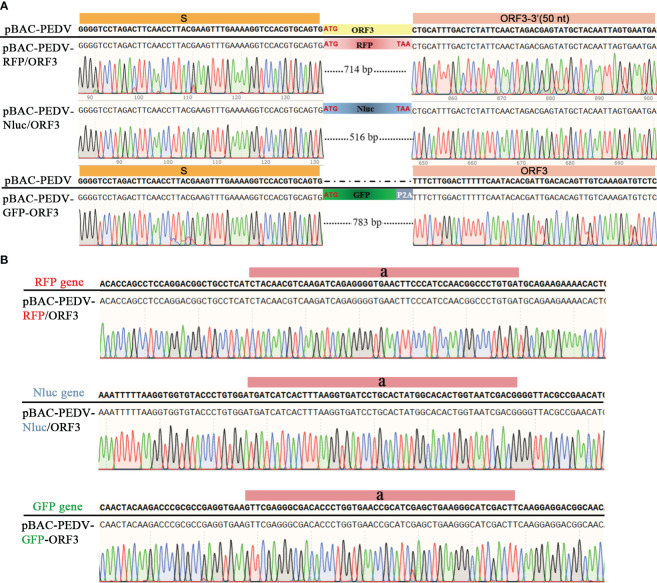
Verification of the RFP, Nluc, or GFP insertion by sanger sequencing of **(A)** first and **(B)** second recombination.

PEDV ORF3 accessory protein is a virporin, which bears a potassium ion channel activity (Wang et al., 2012). It has been reported that reporter genes can replace the non-essential ORF3 gene in PEDV replication (Jengarn et al., 2015). The Red recombination with *E. coli* strain GS1783 is a convenient tool for manipulating virus infectious clones, so three PEDV reporter virus plasmids were generated using the Red recombination. As shown in **Figure 2A**, the ORF3 gene was swapped with a RFP gene or Nluc gene to produce ORF3-deleted variants. To achieve the fusion expression of exogenous genes while preserving the ORF3 gene, the GFP gene and the porcine teschovirus 1 (PTV-1) 2A autocleavage peptide (Caì et al., 2018) were inserted upstream of the PEDV ORF3 gene (**Figure 2A**). The positive co-integrate rate of the first Red recombination, identified through colony PCR, exceeded 80% (**Table 3**). Through twice Red recombination, three reporter virus plasmids were generated and named pBAC-PEDV-RFP/ORF3, pBAC-PEDV-Nluc/ORF3, and pBAC-PEDV-GFP-ORF3, respectively. Then DNA sequencing was performed to verify the first and second recombination of the three resulting plasmids (**Figures 4A, B**). Taken together, three rPEDV reporter virus plasmids were successfully generated with the *E. coli* strain GS1783.

**Table 3:** Recombination rate of first Red recommendation.

Name	Recombination rate of first Red recommendation
pBAC-PEDV-Nluc/ORF3	91.7% (11/12)
pBAC-PEDV-RFP/ORF3	80.0% (12/15)
pBAC-PEDV-GFP-ORF3	81.25% (13/16)

### 3.4 Recovery, identification, and characterization of rPEDV expressing reporter genes

**Figure 5:**
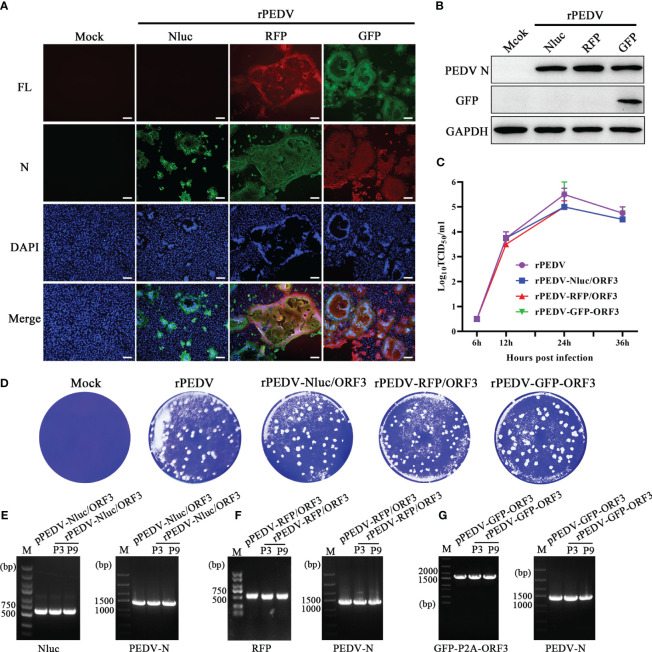
Characterization of the PEDV reporter viruses. **(A)** IFA and **(B)** western blot of mock-, rPEDV-Nluc/ORF3-, rPEDV-RFP/ORF3-, or rPEDV-GFP-ORF3-infected Vero cells at 24 hpi using anti-PEDV N monoclonal antibody. The bar=100 μm. **(C)** Multiple-step growth curves of rPEDV-Nluc/ORF3, rPEDV-RFP/ORF3, and rPEDV-GFP-ORF3 on Vero cells. Cells were infected with rPEDV-Nluc/ORF3, rPEDV-RFP/ORF3, or rPEDV-GFP-ORF3 at an MOI of 0.001. The cell supernatants were collected at 6, 12, 24, or 36 hpi, respectively. The virus titers were determined using a TCID_50_ assay. **(D)** Plaque assay. The confluent Vero cells were infected with rPEDV, rPEDV-Nluc/ORF3-, rPEDV-RFP/ORF3-, or rPEDV-GFP-ORF3 at 0.0001MOIs. At 30 hpi, the cells were stained with 0.5% crystal violet. **(E–G)** The stability of the foreign gene in PEDV reporter virus. The rPEDV-Nluc/ORF3 **(E)**, rPEDV-RFP/ORF3 **(F)** or rPEDV-GFP-ORF3 **(G)** was serially passaged for 9 times in Vero cells. The stability of the foreign genes and the N gene for P3 and P9 virus stocks were amplified using RT-PCR, and the plasmid of the reporter virus was selected as control.

Similar to the strategy in the recovery of rPEDV, we obtained three rPEDV reporter viruses. To furtherly confirm the rPEDV reporter viruses, the expression of PEDV N in rPEDV reporter virus-infected cells was detected by IFA (**Figure 5A**) and western blot (**Figure 5B**). We further examined the growth kinetics of the rPEDV reporter viruses. The results indicated no significant difference in plaque size and morphology (**Figure 5D**). All the recombinant viruses replicated effectively and reached peak titer at 24 hpi (**Figure 5C**). The rPEDV-GFP-ORF3 displayed similar replication kinetics to the rPEDV, while the highest titers of rPEDV-RFP/ORF3 and rPEDV-Nluc/ORF3 were approximately 0.5 logs lower than that of rPEDV. To detect the stability of exogenous genes, rPEDV reporter viruses were serially cultured in the Vero cells. RT-PCR and DNA sequencing analysis showed that the foreign genes (Nluc, RFP, and GFP) were stable in P3 and P9 stocks *in vitro*, and the N gene was as control (**Figures 5E-G**). Taken together, three rPEDV reporter viruses were successfully rescued, and the foreign genes possessed the stability up to P9 in Vero cells.

### 3.5 Growth kinetic of rPEDV-Nluc/ORF3 in different cell lines

**Figure 6:**
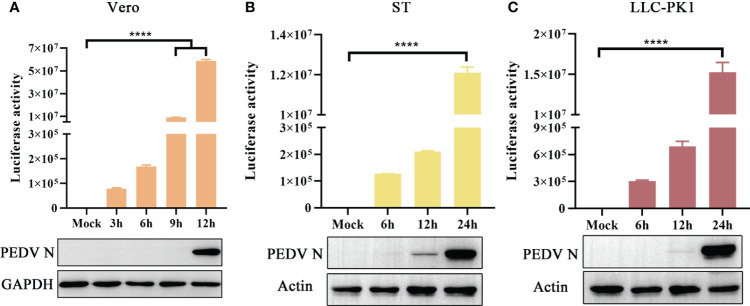
Growth Kinetic of rPEDV-Nluc/ORF3 In Different Cell Lines. Vero cells **(A)**, ST cells **(B)** or LLC-PK1 cells **(C)** were infected with rPEDV-Nluc/ORF3 at an MOI of 0.01. The cell samples were harvested at different time point, and the luciferase activities were detected. The harvested cell lysates were used to western blot with anti-PEDV N monoclonal antibody and GAPDH or Actin. P-values were determined by one-way ANOVA. ****, P < 0.0001.

The novel engineered luciferase, Nluc, is 150 times brighter than firefly or Renilla luciferase. It can produce high-intensity luminescence of the glow-type using the synthetic substrate furimazine while maintaining low background activity (Hall et al., 2012; England et al., 2016). To examine the sensitivity of PEDV infection in different cell lines, Vero, ST, and LLC-PK1 cells were infected with rPEDV-Nluc/ORF3. Subsequently, the cell lysates were collected to detect Nluc activity and PEDV N protein expression levels. The Nluc signal was observed in Vero cells at 3 hpi and exhibited a time-dependent increase in comparison to the mock-infected group. In contrast, western blot assay showed that PEDV N protein expression could be detected at 12 hpi (**Figure 6A**). As shown in **Figures 6B, C**, rPEDV-Nluc/ORF3 could infect both ST and LLC-PK1 cell lines, with the Nluc activity detected early at 6 hpi and PEDV N protein expression observed at 12 hpi. The results indicated that the Nluc reporter virus is more sensitive in quantify features to reflect the viral replication levels than N protein expression, particularly during the early stage of infection.

### 3.6 Application of rPEDV-Nluc/ORF3 in drug screening

**Figure 7:**
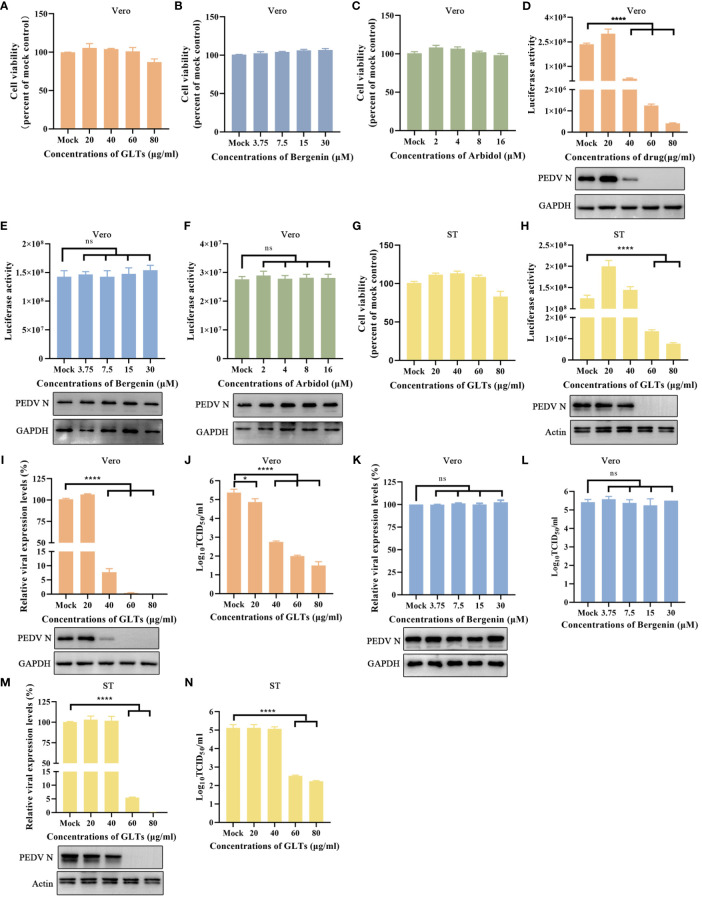
Application of Nluc Reporter Virus in Drug Screening. **(A–C)** GLTs, Bergenin, or Arbidol cytotoxicity in Vero cells were detected with the CCK-8 as described in materials and methods. **(D–F)** The effect of GLTs, Bergenin, or Arbidol on rPEDV-Nluc/ORF3 infected Vero cells by luciferase activity detection and western blot analysis. The cells were pretreated with indicated concentration of the drugs for 2 h and then infected with PEDV Nluc reporter virus for 12 h. Cell lysates were collected for luciferase activity detection and western blot analysis. **(G)** GLTs cytotoxicity in ST cells. **(H)** The effect of GLTs on rPEDV-Nluc/ORF3 infected ST cells by luciferase activity detection and western blot analysis. **(I-L)** The effect of GLTs or Bergenin on PEDV infected Vero cells by qPCR, western blot, and viral titers. **(M, N)** The effect of GLTs on PEDV infected ST cells by real-time PCR, western blot, and viral titers. The cells were pretreated with various concentration of indicated drugs for 2 h and then infected with PEDV for 12 h or 24 h. Cell samples were collected for qPCR and western blot assay. The supernatants were subjected to virus titration using Vero cells. P-values were determined by one-way ANOVA. ns, no significant; *, P < 0.05; ****, P < 0.0001.

To assess the viability of using rPEDV-Nluc/ORF3 for antiviral drug screening, we selected three drugs, namely *Ganoderma lucidum* triterpenoids (GLTs) (Min et al., 1998; Bharadwaj et al., 2019), Bergenin (Yang et al., 2022), and Umifenovir hydrochloride (Arbidol) (Wang et al., 2020). The CCK-8 assay was used to determine the cytotoxic effect of these drugs on Vero cells by measuring cell viability. Results showed there was no significant cytotoxicity on cells when treated with GLTs (up to 80 μg/ml), Bergenin (up to 30 μM), or Arbidol (up to 16 μM) (**Figures 7A–C**). When rPEDV-Nluc/ORF3-infected Vero cells were treated with GLTs (**Figure 7D**), the Nluc activity and expression level of PEDV N protein were dose-dependently inhibited. In comparison, the other two drug treatments (Bergenin and Arbidol) had no significant inhibitory impact on Nluc activity and PEDV N expression in rPEDV-Nluc/ORF3-infected cells (**Figures 7E, F**). These results suggested that GLTs has a significant antiviral effect on rPEDV-Nluc/ORF3 infection. Meanwhile, the antiviral effect of GLTs on rPEDV-Nluc/ORF3 infection in ST cells was also confirmed (**Figures 7G, H**). In addition, the effect of GLTs on wild-type PEDV strain GX4/2021 was further evaluated by qPCR, western blot, and a TCID_50_ assay. The data showed that the infection of PEDV-GX4/2021 was also inhibited by GLTs in a dose-dependent manner (**Figures 7I, J**). The Bergenin treatment was as control (**Figures 7K, L**). When ST cells was treated with GLTs, the replication of wild-type PEDV was also inhibited (**Figures 7M, N**). Taken together, the treatment with GLTs effectively inhibited PEDV infection in Vero and ST cells.

### 3.7 Effect of GLTs after PEDV entry

**Figure 8:**
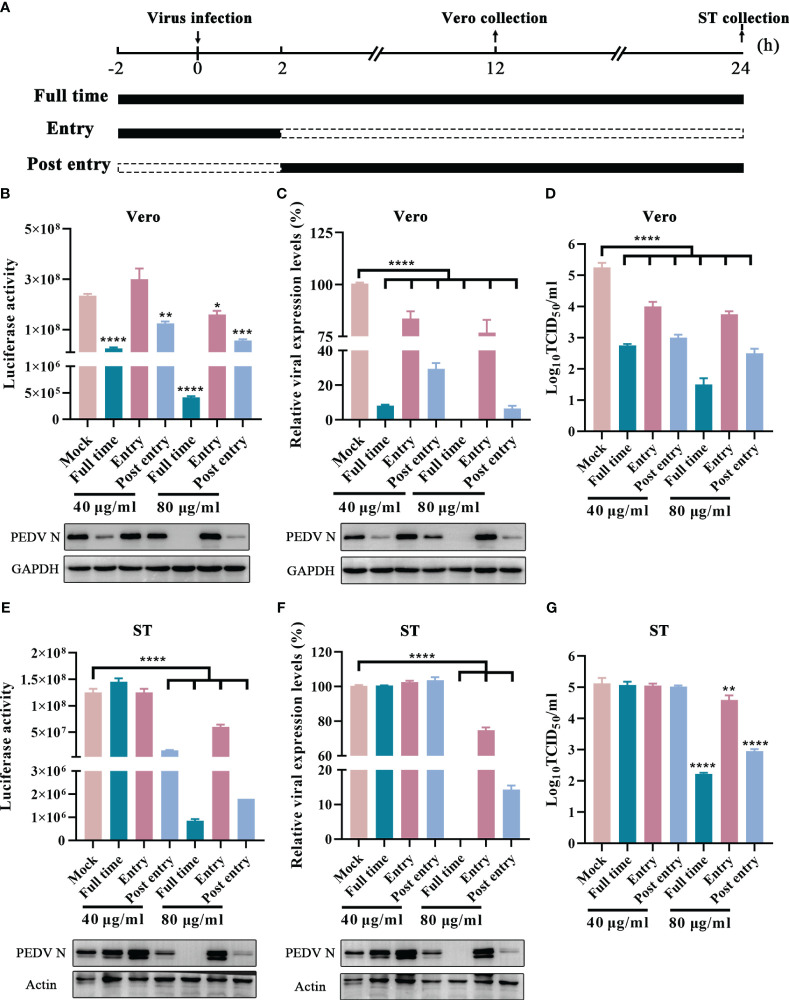
Effect of GLTs after PEDV Entry. **(A)** Schematic diagram of GLTs administration. Before infection, cells of “Entry” and “Full time” groups were incubated with GLTs for 2h. Then the cells were incubated with GLTs and the indicated virus (rPEDV-Nluc/ORF3 or PEDV, MOI=0.01) in the “Entry” and “Full time” groups, while cells in the “Post entry” and positive control groups were treated with the virus only. After 2 h-infection, the media were replaced with fresh medium with GLTs in the “Post entry” and “Full time” groups, while other groups were supplemented with medium without GLTs. Medium of the control groups always contained 0.1% DMSO. The cell samples and the supernatant were collected for evaluation. The effect of GLTs on rPEDV-Nluc/ORF3 infected Vero **(B)** and ST cells **(E)** by luciferase activity detection and western blot analysis. The effect of GLTs on PEDV infected Vero and ST cells by qPCR, western blot analysis **(C, F)** and viral titers **(D, G)**. P-values were determined by one-way ANOVA. *, P < 0.05; **, P < 0.01; ***, P < 0.001; ****, P < 0.0001.

Generally, viral life cycle of coronaviruses includes entry, viral genome translation, RNA synthesis, viral particle assembly, and release, and each stage is crucial to viral infection (V’kovski et al., 2021). To explore the potential inhibition targets, GLTs was added to the cells at varying concentrations during different stages of the virus life cycle, including “Entry”, “Post entry” and “Full time” (**Figure 8A**) (Chen et al., 2022). Compared to the mock-treated groups, the Nluc activity and the expression level of N protein were suppressed in “Post entry” groups of the rPEDV-Nluc/ORF3-infected Vero cells, especially at a concentration of 80 μg/ml (**Figure 8B**). The replication of GX4/2021 strain was also considerably inhibited by GLTs treatment at the stage of “Post entry” as shown by the qPCR and western blot assay (**Figure 8C**). Moreover, viral titers decreased at the virus “Post entry” stage but not at the virus “Entry” stage when compared to control groups in Vero cells (**Figure 8D**). Similar results were obtained when we repeated the experiments in ST cells (**Figures 8E–G**). Taken together, GLTs mainly inhibit PEDV replication after the viruses enter host cells.

## 4 Discussion

A reverse genetics system is a powerful tool for engineering DNA or RNA virus genome. Many infectious cDNA clones for PEDV have been constructed since the first one was reported in 2015 (Jengarn et al., 2015; Beall et al., 2016; Fan et al., 2017; Peng et al., 2020; Zhou et al., 2022). PEDV infectious clone is a valuable molecular tool for creating genetically engineered vaccines and studying the function of viral proteins in virus replication, virulence, and pathogenicity. Although studies on the pathogenesis of PEDV are ongoing, there is a need to establish more platforms for developing effective vaccines and antiviral drugs. In this study, a PEDV infectious cDNA clone based on the BAC system was successfully constructed and the rPEDV was rescued with similar growth kinetics to the wild-type PEDV strain, which could serve as a platform for further investigation. For manipulating the PEDV genome, we used a Red-based technique of the *E. coli* strain GS1783 for the first time. The rate of the first Red recombination is high, which is more than 80%. Furthermore, there are no false-positive results in the Red recombination as validated by Sanger sequence due to the Kana resistance marker cassette. Accordingly, the Red recombination provides a convenient and highly efficient platform for PEDV mutagenesis. Taken together, we have established an efficient reverse genetics system to generate PEDV reporter viruses. Additionally, the Red recombination method described in this study also can be used for other coronaviruses.

Recombinant viruses expressing reporter genes are used by several research groups to perform quantitative analysis of viral replication, identify virus cell receptor, and evaluate MAbs with neutralizing activity against viruses rapidly *in vivo* and *in vitro* (Czakó et al; Nogales et al., 2019; Chiem et al., 2021b; Wang et al., 2021). In addition, the insertion site of reporter gene is important in creating reporter viruses. For coronaviruses, the reporter genes are usually inserted at the position of the accessory protein. The accessory protein ORF7a was replaced with the foreign gene in the rSARS-CoV-2 reporter virus (Chiem et al., 2021a), and the accessory protein NS6 was swapped with a Nluc or GFP gene in the PDCoV reporter virus (Zhang et al., 2020; Fang et al., 2021). In our study, we replaced the only accessory protein in the PEDV genome, ORF3, with reporter gene (Nluc or RFP gene) in the PEDV infectious clone plasmid. Additionally, we constructed a PEDV reporter virus plasmid fused with the ORF3 gene. The plasmid contains the GFP gene and the PTV-1 2A autocleavage peptide inserted upstream of the ORF3 gene. Then, the reporter-expressing rPEDVs were successfully rescued. Our study marks the first time that GFP and PTV-1 2A were inserted upstream of PEDV ORF3 gene, enabling independent expression of GFP through post-translational cleavage by the 2A autocleavage peptide (**Figure 5B**). The foreign genes (Nluc, RFP, and GFP) in reporter-expressing rPEDV were stable to culture up to P9 in Vero cells, indicating the PEDV genome could at least accommodate the foreign gene with the size of GFP gene at the insertion site of ORF3 gene. These foreign genes were chosen not only for the distinctive fluorescent properties (RFP and GFP) but also for the smaller size (Nluc) than firefly (Photinus pyralis) or Renilla luciferase and high physical stability (Hall et al., 2012). The rPEDV expressing fluorescent protein (RFP or GFP) could be applied to easily monitor viral infection in cultured cells. Consistent with the study reported (Tran et al., 2013; Fang et al., 2021; Li et al., 2021; Ye et al., 2021), our results also showed that Nluc activity was more readily detectable than the expression of viral N protein in the cells infected with Nluc-tagged reporter virus. This confirms that reporter viruses can effectively evaluate viral replication. Then, the rPEDV-Nluc/ORF3 was used as a tool to screen anti-PEDV drugs.

*Ganoderma lucidum* (*G. lucidum*), an oriental fungus, has been used for health promotion and treatment of various diseases in Asia (Cilerdžić et al., 2014; Wang and Lin, 2019). GLTs is a category of the main bioactive and medicinal components in *G. lucidum*. The GLTs exhibit various pharmacological effects, such as anticancer (Wu et al., 2013), anti-atherosclerosis (Hsu et al., 2018), anti-hyperlipidemia (Guo et al., 2020), and antivirus (Ahmad et al., 2021). Bergenin is a bioactive compound derived from many medicinal plants. Bergenin shows hepatoprotective (Xiang et al., 2020), antioxidant (Zhang et al., 2022a), anti-arthritic (El-Hawary et al., 2016), antidiabetic (Kumar et al., 2012), anticancer (Shi et al., 2021), and antiviral activity (Tomimori, 2000). Umifenovir hydrochloride (Arbidol) is a broad-spectrum antiviral drug, which could effectively resist Influenza A Virus and SARS-CoV-2 *in vitro* (Wang et al., 2020; Li et al., 2022). To date, the antiviral activity of the three drugs on PEDV has not been reported as yet. Among the reported compounds with anti-PEDV activity, epigallocatechin-3-gallate inhibited PEDV infection during the viral life cycle of attachment, entry, replication, and assembly (Huan et al., 2021), and A77 1726 could restrict PEDV replication by resisting Janus kinases and Src kinase activity (Li et al., 2020). In our study, the results showed that GLTs could effectively inhibit PEDV replication, as well as other PEDV strains (see **Figure S1** in the **Supplemental Material**), particularly after the stage of virus “Entry”. The mechanism of the antiviral drugs is usually through targeting the virus itself or the important host factor related to the viral life cycle. However, the specific antiviral mechanism of GLTs is not clear. Recent studies suggest that GLTs could act as inhibitors of NS2B-NS3 protease in DENV infection (Bharadwaj et al., 2019). Thus, we suspect that GLTs may inhibit PEDV replication by targeting protease activity such as 3CL protease to inhibit PEDV replication; however, it requires further investigation.

In summary, we have successfully constructed a PEDV full-length cDNA clone with the BAC system. Three reporter-expressing rPEDVs, including RFP, Nluc, or GFP gene, were generated using Red recombination with the *E. coli* strain GS1783, building a convenient and efficient method for engineering the genomes of PEDV and other coronaviruses. We found that the replication of PEDV was effectively inhibited by the GLTs using the Nluc-tagged PEDV reporter virus (rPEDV-Nluc/ORF3). We believe that this study lays the foundation for investigating the pathogenic mechanism of PEDV and developing genetic engineering vaccines.

## Data availability statement

The raw data supporting the conclusions of this article will be made available by the authors, without undue reservation.

## Author contributions

SP: Formal analysis, Investigation, Methodology, Writing – original draft. CM: Conceptualization, Data curation, Formal analysis, Investigation, Methodology, Project administration, Validation, Writing – original draft. ZC: Conceptualization, Funding acquisition, Project administration, Supervision, Writing – review & editing.
