# Supplementary material for: Red recombination enables a wide variety of markerless manipulation of porcine epidemic diarrhea virus genome to generate recombinant virus
Source: Front Cell Infect Microbiol. 2024 Jan 22;13:1338740. doi: 10.3389/fcimb.2023.1338740 (PMC10839022; doi:10.3389/fcimb.2023.1338740)
Supplement: Supplementary file 1 [file DataSheet_1.docx]

Fig. S1


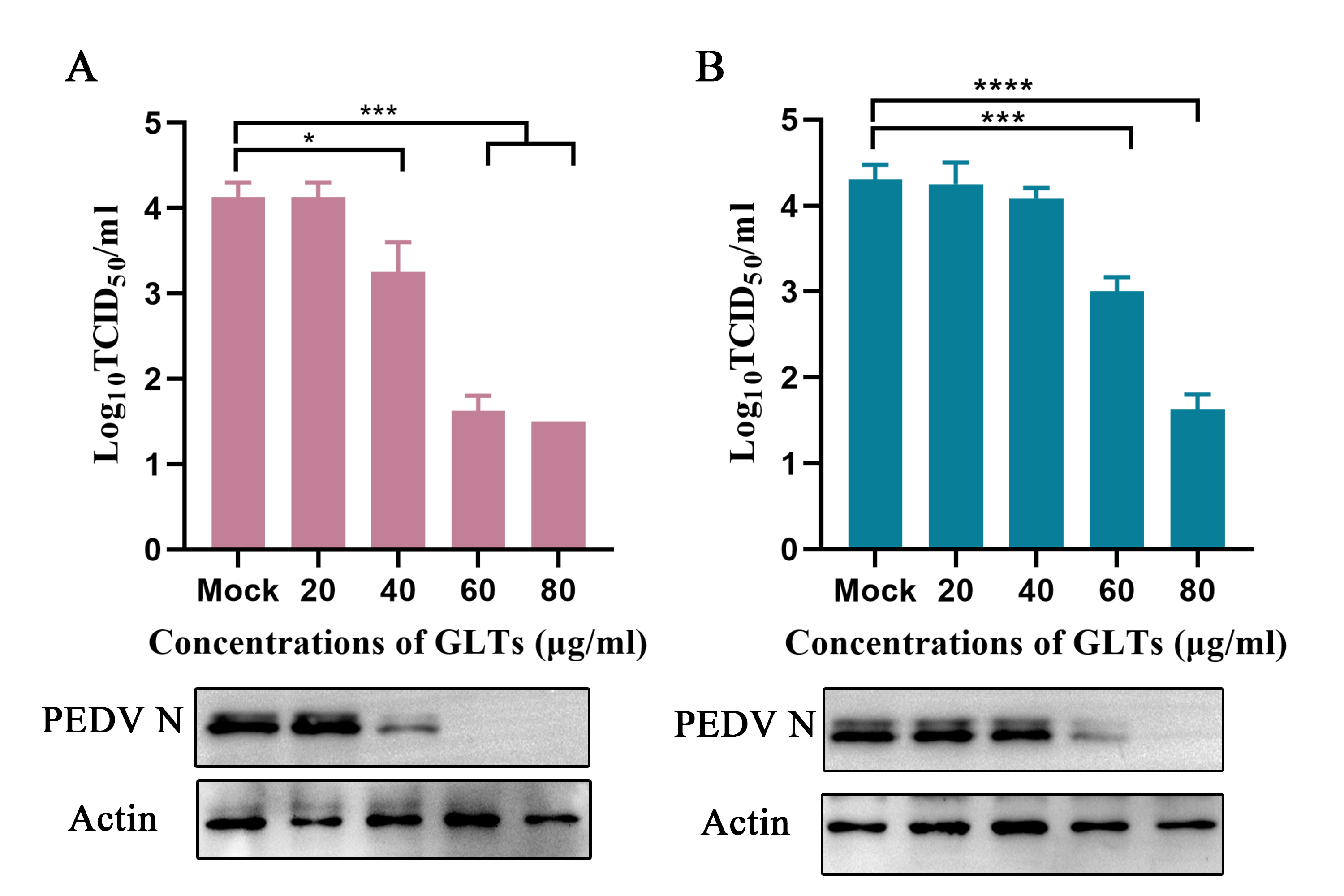


**Figrue S1. Effect of GLTs on other PEDV strains.** The effect of GLTs on PEDV-GX6/2021 (GenBank accession number OP603897) (A) and PEDV-GX01/2022 (GenBank accession number OQ731917) (B) infected Vero cells by viral titers and Western blot analysis.

Table S1 Primers used for Red Recombination

| b-PEDV-RFP F | GGGGTCCTAGACTTCAACCTTAC |
| --- | --- |
| PEDV-RFP-c R | TCATTCACTAATTGTAGCATACTCGTCTAG |
| b-PEDV-Nluc F | GGGGTCCTAGACTTCAACCTTAC |
| PEDV-Nluc-c R | TCATTCACTAATTGTAGCATACTCGTCTAG |
| b-PEDV-GFP F | GGGGTCCTAGACTTCAACCTTACGAAGTTTGAAAAGGTCCACGTGC |
| PEDV-GFP-c R | TCATTCACTAATTGTAGCATACTCGTCTAGTTGAATAGAGTCAAATG |
| PEDV-S-GFP F | GTTTGAAAAGGTCCACGTGCAGTGATGGTGAGCAAGGGCGAGGAG |
| PE-GFP-a-ISceI R | CAATTACCCTGTTATCCCTATCCTCCTTGAAGTCGATGCCC |
| PE-GFP-a F | AAGGCAGTTATTGGTGCCCTTAAACGCCGTTCGAGGGCGACACCCTG |
| P2A-PE-ORF3 R | ATCGTGTATTGAAAAAGTCCAAGAAACGGTCCAGGATTCTCTTCGAC |
| P2A-PE-ORF3 R2 | GAGACATCTTTGACAACTGTGTCAATCGTGTATTGAAAAAGTCCAAG |
| ORF R | GACGAGTATGCTACAATTAGTGAATGA |
| I-SceI-CATpro F | TAGGGATAACAGGGTAATTGATCGGCACGTAAGAGGTTCC |
| Kan R | GTTTAAGGGCACCAATAACTGCCTTAAAAAAATTAGAAAAACTCATCGAGCATCAAATG |
| PEDV-S-Nluc F | GTTTGAAAAGGTCCACGTGCAGTGATGGTCTTCACACTCGAAGATTTCGTTG |
| PE-Nluc-a-ISceI R | CAATTACCCTGTTATCCCTACGTCGATTACCAGTGTGCCA |
| PE-Nluc-a F | TATTGGTGCCCTTAAACGCCTGATCATCACTTTAAGGTGATCCTGC |
| Nluc R | TAGTTGAATAGAGTCAAATGCAGTTACGCCAGAATGCGTTCGCAC |
| PEDV-S-GFP F2 | GGGGTCCTAGACTTCAACCTTACGAAGTTTGAAAAGGTCCACGTGC |
| PEDV-Nluc-ORF3 R2 | TCATTCACTAATTGTAGCATACTCGTCTAG |
| PEDV-S-RFP F | GTTTGAAAAGGTCCACGTGCAGTGATGGTGTCTAAGGGCGAAGAGCTG |
| PE-RFP-a-ISceI R | ATCAATTACCCTGTTATCCCTATCACAGGGCCGTTGGATGG |
| PE-RFP-a F | GTTATTGGTGCCCTTAAACGCCCTACAACGTCAAGATCAGAGGGG |
